# A Friend

**Published:** 1864-06

**Authors:** 


A FRIEND who has recently passed a successful examination at Washington, qualifying him for the rank of Surgeon of Volunteers, gives us the following account of his examination:

"The examination commenced on Monday, and was continued every day through the week. Four days of written replies to questions, the candidates being placed in a room, with writing materials *et preterea nihil*, except an orderly to prevent communications. Fifth day, each candidate taken alone by one of the commission of three. Oral examination. Sixth day, at Douglas Hospital, in the wards and dead-house. Operations on the subject. The object of the Board seemed to be not merely to ascertain whether the candidates knew enough to be a Surgeon or Assistant Surgeon of Volunteers, but to discover the whole extent of his attainments. In anatomy, chemistry, physiology, pathology, his point of saturation (so to speak), was reached. This you will see can be readily done by a division of subjects among the members of the Board. I should have said that an autobiography is required and a disclaimer of any connection at any time with any form of irregular practice. With all this you will see that the Government are put in possession of the full professional value of each of its medical officers of this corps."

